# Prevalence and determinants of obesity among primary school children in Dar es Salaam, Tanzania

**DOI:** 10.1186/0778-7367-71-26

**Published:** 2013-10-07

**Authors:** Alfa J Muhihi, Rose N M Mpembeni, Marina A Njelekela, Amani Anaeli, Omary Chillo, Sulende Kubhoja, Benjamin Lujani, Mwanamkuu Maghembe, Davis Ngarashi

**Affiliations:** 1 Africa Academy for Public Health, Dar es Salaam, Tanzania; 2 Ifakara Health Institute, Ifakara, Morogoro, Tanzania; 3 Department of Epidemiology and Biostatistics, Muhimbili University of Health and Allied Sciences, Dar es Salaam, Tanzania; 4 Department of Physiology, Muhimbili University of Health and Allied Sciences, Dar es Salaam, Tanzania; 5 Muhimbili National Hospital, Dar es Salaam, Tanzania; 6 Department of Development Studies, Muhimbili University of Health and Allied Sciences, Dar es Salaam, Tanzania; 7 Department of Pediatrics and Child Health, Muhimbili University of Health and Allied Sciences, Dar es Salaam, Tanzania

**Keywords:** Prevalence, Child obesity, Determinants, Urban, Children, Tanzania

## Abstract

**Background:**

Childhood obesity has increased dramatically and has become a public health concern worldwide. Childhood obesity is likely to persist through adulthood and may lead to early onset of NCDs. However, there is paucity of data on obesity among primary school children in Tanzania. This study assessed the prevalence and determinants of obesity among primary school children in Dar es Salaam.

**Methods:**

A cross sectional study was conducted among school age children in randomly selected schools in Dar es Salaam. Anthropometric and blood pressure measurements were taken using standard procedures. Body Mass Index (BMI) was calculated as weight in kilograms divided by the square of height in meters (kg/m^2^). Child obesity was defined as BMI at or above 95th percentile for age and sex. Socio-demographic characteristics of children were determined using a structured questionnaire. Logistic regression was used to determine association between independent variables with obesity among primary school children in Dar es Salaam.

**Results:**

A total of 446 children were included in the analysis. The mean age of the participants was 11.1±2.0 years and 53.1% were girls. The mean BMI, SBP and DBP were 16.6±4.0 kg/m^2^, 103.9±10.3mmHg and 65.6±8.2mmHg respectively. The overall prevalence of child obesity was 5.2% and was higher among girls (6.3%) compared to boys (3.8%). Obese children had significantly higher mean values for age (p=0.042), systolic and diastolic blood pressures (all p<0.001). Most obese children were from households with fewer children (p=0.019) and residing in urban areas (p=0.002). Controlling for other variables, age above 10 years (AOR=3.3, 95% CI=1.5-7.2), female sex (AOR=2.6, 95% CI=1.4-4.9), urban residence (AOR=2.5, 95% CI=1.2-5.3) and having money to spend at school (AOR=2.6, 95% CI=1.4-4.8) were significantly associated with child obesity.

**Conclusions:**

The prevalence of childhood obesity in this population was found to be low. However, children from urban schools and girls were proportionately more obese compared to their counterparts. Primary preventive measures for childhood obesity should start early in childhood and address socioeconomic factors of parents contributing to childhood obesity.

## Background

The increasing prevalence of childhood obesity has become a growing matter of public health concern worldwide. Obesity has increased from 4.2% in 1990 to 6.7% in 2010 worldwide and is expected to reach 9.1% in 2020 [1]. The risk is more for children in industrialized countries where the prevalence has increased more than twice in the past three decades [2]. In 2010, 43 million children were estimated to be obese worldwide, out of whom, 35 million (81.4%) were from developing countries [1]. The estimated prevalence of childhood obesity in Africa in 2010 was 8.5% and is expected to reach 12.7% in 2020 [1]. North Africa is the region with highest prevalence of childhood obesity in Africa [3].

Few studies have been conducted on the prevalence of obesity among primary school children in Tanzania [4-6]. In a study conducted in Dodoma and Kinondoni, Mosha et al. showed that the prevalence of obesity among children aged 6–9 years was 5.6% and 6.3% respectively [5]. A similar low prevalence of child obesity (5.3%) was also reported by Chillo et al. in a study conducted in Dar es Salaam and Morogoro regions [4].

Genetic and environmental factors have been documented as potential causes of obesity [7,8]. The rising prevalence of childhood obesity in developing countries is attributed to the growing urbanization, transition towards high caloric western diet of refined and fast foods and sedentary lifestyle [9,10]. Tanzania is not spared by the consequences of globalization, and profound societal changes have taken place especially in urban settings [11,12]. Leisure time activities are increasingly sedentary. Entertainments such as television, video and computer games are also widely and easily accessible. Hours spent on viewing television and computer usage is associated with increasing BMI among children [13]. Walking to and from school and morning joggings provided a potentially important opportunity for establishing daily physical activity among primary school children [14].

Literature from both, developed and developing countries have documented association between childhood obesity with many adverse health effects, ranging from hyperlipidemia, hypertension, respiratory disorders, glucose intolerance and type 2 diabetes mellitus, depression and low self-esteem to social discrimination [15-19]. All these adverse effects point to the necessity of preventing childhood obesity.

With the increasing body of evidence that childhood obesity often persist through adulthood [20-22] and higher possibility of lifestyle modification in children as opposed to adults, interventions aiming at modifying risk factors to prevent childhood obesity should be a top priority [23]. It is thus imperative to understand the magnitude and determinants of childhood obesity in order to develop effective preventive strategies. The present study assessed the prevalence and determinants of childhood obesity among primary school children in Dar es Salaam.

## Methods

### Study design, site and population

This cross-sectional study was conducted among primary school children aged 6–17 years from nine primary schools in three districts of Ilala, Kinondoni and Temeke in Dar es Salaam region. A sampling frame of all public and private schools in Dar es Salaam region was obtained from the district education authorities. Multistage cluster sampling was used to select schools to be involved in the study. Selection was made to ensure equal representation primary schools from both urban and rural settings of Dar es Salaam. Within each selected school one class (from class 1–7) was randomly selected and all children from the selected class were invited to participate into the study. Consent forms containing detailed information about the study were given to children to take to their parents. Signed consent forms were returned by the children on the scheduled day for data collection. Study questionnaires with both closed and open-ended questions were used to gather the required information from the participants.

### Anthropometric measurements

Anthropometric measurements were conducted by trained research assistants early in the morning before classes began. Measurements were conducted in a dedicated room at each school with children wearing light clothes and with no shoes. Body weight was measured using a self-calibrating precision digital scale (Omron, Japan). Height was measured to the nearest 0.1 cm by a fixed Shorr measuring board (Shorr Productions, Olner, MD). BMI was then calculated as weight in kilograms divided by the square of height in meters (kg/m^2^). All measurements were taken while observing standard precautions [24]. Child obesity was defined based on BMI percentiles for age and sex. Children with BMI at or above 95th percentile for age and sex were considered obese [25].

### Blood pressure measurements

Blood pressure was measured using a standardized digital blood pressure measuring machine (Omron Digital HEM-907, Tokyo, Japan). Blood pressure readings were taken after the child had rested for at least 5–10 minutes. Blood pressure was measured on the left upper arm with the child in a seated position. Three readings were taken for each participant, and an average of the three readings was used during analysis.

### Sociodemographic information

A structured questionnaire was used to collect socio-demographic information from the participating children. Important sociodemographic information was obtained from parents/guardian during the interview. Education level of the parents/guardian was assessed and categorized as; no formal education (can neither read nor solve simple mathematics), primary education (attended school for up to 7 years), secondary education (attended school for 12–14 years) and college/university education (attended university or attended school for 12 years and college training for 1 or more years. Parents’/guardians’ main occupations were grouped as housewife, business, farmer and government employee.

### Statistical analysis

Data analysis ranged from descriptive statistics (means, standard deviations and frequencies) describing the general characteristics to identification of statistically significant associations. Continuous variables were compared using t-test or Mann–Whitney U test as appropriate while categorical variables were compared using chi-square (*X*^*2*^) test or fisher’s exact test. Odds ratios (OR) and their 95% confidence interval (CI) were estimated in univariate and multivariate analysis to measure the association of independent variables and child obesity. All statistical analyses were performed using Statistical Analysis Software (SAS 9.2, Institute Inc., North Carolina, USA) and values with p-value ≤ 0.05 were considered statistically significant.

### Ethical issues

Ethical clearance was obtained from the Research Ethics Review Committee of Muhimbili University of Health and Allied Sciences. An informed consent to participate into the study was obtained from the parents/guardians of the selected children prior to the study. Confidentiality of information was adhered to by using unique identification numbers on the data collection tools instead of participant names. Questionnaires were stored in locked cabinets at MUHAS and were accessible to the researchers only.

## Results

### Socio-demographic characteristics of the study population

A total of 446 children from nine primary schools in Dar es Salaam were included into the study. The mean age of the children was 11.1±2.0 years (11.3±2.1 years for boys and 11.1±2.0 years for girls). The mean BMI for girls (17.0±4.0 kg/m^2^) was higher than that of boys (16.1±4.0 kg/m^2^), and this difference was significant (p=0.002). Statistically significant gender difference was also observed for education level of the mother (p=0.014) (Table 1).

**Table 1:** Socio-demographic characteristics of primary school children in Dar es Salaam, Tanzania 2011

**Variable**	**All (N=446)**		**Boys (N=209)**		**Girls (N=237)**		**P-value**
	**Mean±SD**	**(n)%**	**Mean±SD**	**(n)%**	**Mean±SD**	**(n)%**	
Age in years	11.1±2.0		11.3±2.1		11.1±2.0		0.591
Place of residence							0.294
Rural		197 (44.2)		98 (46.9)		99 (41.8)	
Urban		249 (55.8)		111 (53.1)		138 (58.2)	
Religion							0.777
Christian		225 (50.4)		107 (51.2)		118 (49.8)	
Muslim		221 (49.6)		102 (48.8)		119 (50.2)	
Number of adults (≥18yrs)	6.2±2.2		6.3±2.3		6.2±2.0		0.949
Number of children (<18yrs)	3.2±2.1		3.0±1.6		3.3±2.5		0.307
Education level of mother							0.014
No formal		27 (6.3)		11 (5.5)		16 (6.9)	
Primary		144 (33.3)		67 (33.3)		77 (33.3)	
Secondary		106 (24.5)		46 (22.9)		60 (26.0)	
College/University		68 (15.8)		44 (21.9)		24 (10.4)	
No response		87 (20.1)		33 (16.4)		54 (23.4)	
Occupation of mother							0.188
Housewife		86 (20.2)		33 (16.8)		53 (23.1)	
Business		183 (43.0)		82 (41.6)		101 (44.1)	
Farmer		59 (13.8)		33 (16.8)		26 (11.4)	
Government employee		39 (9.2)		17 (8.6)		22 (9.6)	
Other		59 (13.8)		32 (16.2)		27 (11.8)	
SBP	103.9±10.3		103.4±10.4		104.3±10.2		0.111
DBP	65.6±8.2		66.4±8.3		66.7±8.2		0.433
BMI	16.6±4.0		16.1±4.04		17.0±4.0		0.002

### Prevalence, distribution and characteristics of obese children

Table 2 shows the prevalence of obesity in the population of primary school children in Dar es Salaam. The overall prevalence of child obesity was 5.2%. Obesity was higher among girls (6.3%) compared to boys (3.8%). Similarly, the prevalence of overweight was higher among girls (13.1%) compared to boys (6.3%). The distribution of BMI categories among girls and boys was statistically significant (p=0.031).

**Table 2:** Prevalence of obesity among primary school children in Dar es Salaam, Tanzania 2011

**Characteristic**	**All (N=446)**	**Boys (209)**	**Girls (N=237)**	**P-value**
BMI category				
Underweight	65 (14.6)	36 (17.2)	29 (12.2)	
Normal weight	314 (70.4)	152 (72.7)	162 (68.4)	0.031
Overweight	44 (9.8)	13 (6.3)	31 (13.1)	
Obese	23 (5.2)	8 (3.8)	15 (6.3)	

Table 3 shows the distribution and characteristics of obese children. More girls were obese than boys with 6.3% and 3.8% respectively. The mean systolic and diastolic blood pressures were significantly higher among obese compared to non-obese children (all p<0.001). The mean BMI was significantly higher among children from households with fewer number of children (p=0.019). With regard to area of residence, children living in urban areas of Dar es Salaam were more than 5 times more likely to be obese compared to their counterparts living in rural settings (p=0.002).

**Table 3:** Distribution and characteristics of obese and non-obese primary school children in Dar es Salaam, Tanzania 2011

**Variable**	**Non-obese children**		**Obese children**		**P-value**
	**Mean±SD**	**(n)%**	**Mean±SD**	**(n)%**	
Age in years	11.2±2.1		12.0±1.2		0.042
Gender					
Boys		201 (96.2)		8 (3.8)	0.286
Girls		222 (93.7)		15 (6.3)	
Place of residence					
Rural		194 (98.5)		3 (1.5)	0.002
Urban		229 (92.0)		20 (8.0)	
Religion					
Christian		211 (93.8)		14 (6.2)	0.393
Muslim		212 (95.9)		9 (4.1)	
Number of adults (≥18yrs)	6.3±2.2		5.7±1.4		0.379
Number of children (<18yrs)	3.2±2.2		2.3±1.0		0.019
Education level of mother					0.713
No formal		26 (96.3)		1 (3.7)	
Primary		139 (96.5)		5 (3.5)	
Secondary		99 (93.4)		7 (6.6)	
College/University		63 (92.7)		5 (7.3)	
No response		82 (94.3)		5 (5.7)	
Occupation of mother					0.217
Housewife		78 (90.7)		8 (9.3)	
Business		176 (96.2)		7 (3.8)	
Farmer		58 (98.3)		1 (1.7)	
Government employee		36 (92.3)		3 (7.7)	
Other		56 (94.9)		3 (5.1)	
SBP	103.4±9.9		114.3±11.8		<0.001
DBP	66.0±7.9		75.8±9.7		<0.001

### Socio-demographic factors associated with childhood obesity

Tables 4 and 5 present results for univariate and multivariate analyses between various independent factors associated with childhood obesity. Children aged above 10 years were 3 times likely to be obese (COR=2.9; 95% CI: 1.4-5.9). Girls had twice the risk of being obese compared to boys (COR=2.2; 95% CI: 1.2-3.7). Urban residence was associated with a three-fold increase in the risk of being obese (COR=2.9, 95% CI: 1.6 – 5.3) and having money to spend at school also doubled the risk of being obese (COR=2.2, 95% CI: 1.3 – 3.8).

**Table 4:** Univariate analysis for predictors of obesity among primary school children in Dar es Salaam, Tanzania 2011

**Independent variables**	**Non-obese children N (%)**	**Obese children N (%)**	**COR (95% CI)**
Age (years)			
10 years or less	136 (98.5)	2 (1.5)	1.0
Above 10 years	287 (93.2)	21 (6.8)	2.9(1.4 – 5.9)^§^
Gender			
Boys	201 (96.2)	8 (3.8)	1.0
Girls	222 (93.7)	15 (6.3)	2.2 (1.2 – 3.8)^§^
Area of residence			
Rural	194 (98.5)	3 (1.5)	1.0
Urban	229 (92.0)	20 (8.0)	2.9 (1.6 – 5.3)^§^
Education level of mother			
No formal	26 (96.3)	1 (3.7)	1.0
Primary	139 (96.5)	5 (3.5)	0.3 (0.1 – 1.1)
Secondary	99 (93.4)	7 (6.6)	0.8 (0.3 – 2.3)
College/University	63 (92.6)	5 (7.4)	0.6 (0.2 – 1.9)
Declined response	82 (94.3)	5 (5.7)	0.6 (0.2 – 1.7)
Occupation of mother			
Housewife	78 (90.7)	8 (9.3)	1.0
Business	176 (96.2)	7 (3.8)	0.7 (0.4 – 1.5)
Farmer	58 (98.3)	1 (1.7)	0.7 (0.3 – 1.9)
Government employee	36 (92.3)	3 (7.7)	1.4 (0.6 – 3.6)
Other	56 (94.9)	3 (5.1)	0.5 (0.2 – 1.5)
Money to spend at school/day			
≤500 Tshs	306 (97.1)	9 (2.9)	1.0
>500 Tshs	117 (89.3)	14 (10.7)	2.2 (1.3 – 3.8)^§^
Religion			
Christian	211 (93.8)	14 (6.2)	1.0
Muslim	212 (95.9)	9 (4.1)	1.2 ( 0.7 – 2.1)
Number of adults in the family			
≤4	79 (91.7)	7 (8.3)	1.0
5 to 7	249 (94.3)	15 (5.7)	0.8 (0.4 – 1.5)
>7	95 (99.0)	1 (1.0)	0.6 (0.2 – 1.3)
Number of children in the family			
≤2	185 (92.0)	16 (8.0)	1.0
3-5	195 (96.5)	7 (3.5)	0.7 (0.4 – 1.2)
>5	43 (100.0)	0 (0.0)	0.3 (0.1 – 1.1)

§ = p-value <0.05.

COR = Crude Odds Ratio.

CI = Confidence Interval.

**Table 5:** Multivariate logistic regression models for independent predictors of obesity among primary school children in Dar es Salaam, Tanzania 2011

**Independent variables**	**Non-obese children N (%)**	**Obese children N (%)**	**AOR (95% CI)**
Age (Yrs)			
10 years or less	136 (98.5)	2 (1.5)	1.0
More than 10 years	287 (93.2)	21 (6.8)	3.3 (1.5 – 7.2)^§^
Gender			
Boys	201 (96.2)	8 (3.8)	1.0
Girls	222 (93.7)	15 (6.3)	2.6 (1.4 – 4.9)^§^
Area of residence			
Rural	194 (98.5)	3 (1.5)	1.0
Urban	229 (92.0)	20 (8.0)	2.5 (1.2 – 5.3)^§^
Education level of mother			
No formal	26 (96.3)	1 (3.7)	1.9 (0.5 – 7.2)
Primary	139 (96.5)	5 (3.5)	0.5 (0.2 – 1.4)
Secondary	99 (93.4)	7 (6.6)	0.9 (0.4 – 2.3)
College/University	63 (92.6)	5 (7.4)	1.0
Declined response	82 (94.3)	5 (5.7)	0.6 (0.2 – 1.8)
Occupation of mother			
Housewife	78 (90.7)	8 (9.3)	1.0 (0.3 – 3.0)
Business	176 (96.2)	7 (3.8)	0.7 (0.3 – 2.1)
Farmer	58 (98.3)	1 (1.7)	0.9 (0.3 – 3.3)
Government employee	36 (92.3)	3 (7.7)	1.0
Other	56 (94.9)	3 (5.1)	0.6 (0.2 – 2.1)
Money spent at school/day			
≤500 Tshs	306 (97.1)	9 (2.9)	1.0
>500 Tshs	117 (89.3)	14 (10.7)	2.6 (1.4 – 4.8)^§^
Religion			
Christian	211 (93.8)	14 (6.2)	1.0
Muslim	212 (95.9)	9 (4.1)	1.2 ( 0.6 – 2.2)
Number of adults in the family			
≤4	79 (91.7)	7 (8.3)	1.0
5 to 7	249 (94.3)	15 (5.7)	0.9 (0.4 – 1.9)
>7	95 (99.0)	1 (1.0)	0.9 (0.4 – 2.5)
Number of children in the family			
≤2	185 (92.0)	16 (8.0)	1.0
3-5	195 (96.5)	7 (3.5)	0.5 (0.3 – 1.0)
>5	43 (100.0)	0 (0.0)	0.3 (0.1 – 1.1)

§ = P-value <0.05.

CI = Confidence Interval.

AOR = Adjusted Odds Ratio.

After adjusting for all independent variables, age above 10 years (AOR=3.3, 95% CI=1.5-7.2), female sex (AOR=2.6, 95% CI=1.4-4.9), urban residence (AOR=2.5, 95% CI=1.2-5.3) and having money to spend at school (AOR=2.6, 95% CI=1.4-4.8) were significant independent determinants of obesity in this population of primary school children.

## Discussion

This study has established the prevalence and determinants of obesity in a representative sample of primary school children in a large business capital of Dar es Salaam. Our results showed that the prevalence of obesity was low (5.2%). After adjusting for other independent variables, age above 10 years, female gender, urban residence and having money to spend at school were the most significant independent determinants of obesity in this population of primary school children.

The prevalence of child obesity found in this study was comparable to that found in previous studies conducted in Tanzania [4,5], and slightly higher compared to that reported from South Africa [26]. Higher prevalence of child obesity has been reported from North Africa [27], and other developing [28,29] and developed countries [30-33]. In this study, prevalence of child obesity was higher among girls than boys. Other studies conducted among children in Africa [26,27,34] and outside Africa [35] have reported similar gender difference in the prevalence of child obesity.

Findings on the association between socioeconomic status with childhood obesity have been controversial, with some studies reporting a positive association of lower education and lower socio-economic status with obesity [9,36]. Other studies have demonstrated that family’s higher socioeconomic status, increased parental education and high household income to be associated with increased risk of childhood obesity [27,37]. People in the higher socioeconomic strata in the population were the most affected when obesity emerged in developing countries [38]. Our findings have indicated that higher socioeconomic status as shown by higher amount of money given to a child to spend at school per day was associated with increased risk of obesity. Giving child money to spend at school increases the chance of the child to buy fast foods like French fries and sweetened snack like biscuits while at school and consequently, they are predisposed to higher risk of child obesity. Frequent snacking has been reported to be associated with BMI and BMI changes [39].

Our study did not find significant relationship between level of education and occupation of the mother with risk of obesity in children. Our findings appear to differ from conclusions reached by other studies which reported increased risk of overweight among children of mothers with higher education [34]. Contrary to findings from Ghana which documented an association between maternal employment and childhood overweight among advantaged households [40], an Indian study reported maternal unemployment to be a risk factor for childhood overweight [41]. We also had data on religion, number of children and adults in a family, and none of these variables was significantly related to child obesity.

Another interesting finding from our present study was that obese children had significantly higher systolic and diastolic blood pressure. Other studies have also reported similar findings of association between obesity and increased risk of hypertension [42,43]. Although our study did not assess the association between obesity with other health outcomes, there is an increasing body of literature showing that childhood obesity is associated with many other adverse health effects including hyperlipidemia, respiratory disorders, glucose intolerance and type 2 diabetes mellitus, depression and low self-esteem [15,16,19].

Our study has several limitations. The study did not assess many other factors that influence the risk of childhood obesity. Weight gain during pregnancy, maternal obesity and birth weight have been shown to be strongly associated with childhood obesity [8,22,29]. Unhealthy dietary pattern and physical inactivity are important factors impacting on the risk of obesity in children [37,44,45]. Our study did not gather data to assess the relationship of these variables with the risk of childhood obesity. It is also possible that other unidentified confounders such as genetic factors may have influenced the findings of our study.

## Conclusions

In conclusion, the findings of this study have shaded light on the prevalence and determinants of obesity among primary school children in Tanzania. The prevalence of obesity in this population was found to be low. However, children from urban schools and girls had proportionately higher rates of obesity compared to their counterparts. We recommend further studies to increase our understanding of the prenatal, perinatal and postnatal predictors of childhood obesity. Because early interventions on modifiable risk factors are likely to decrease the rate of childhood obesity, educational programs about obesity and associated health consequences should start early in childhood so as prevent the increasing prevalence of childhood obesity in Tanzania.

## Competing interests

The authors declare that they have no competing interests.

## Authors’ contributions

AJM analyzed and interpreted the data, drafted and revised the manuscript. RNM analyzed data and critically reviewed the manuscript. MAN conceived and designed the study, participated in data collection and critically reviewed the manuscript. AA OC SK MM and DN participated in data collection and reviewed the manuscript. BL took anthropometric measurements and reviewed the manuscript. All authors read and approved the final manuscript version.
